# Metabolic engineering of *Ashbya gossypii* for deciphering the de novo biosynthesis of γ-lactones

**DOI:** 10.1186/s12934-019-1113-1

**Published:** 2019-03-28

**Authors:** Rui Silva, Tatiana Q. Aguiar, Eduardo Coelho, Alberto Jiménez, José Luis Revuelta, Lucília Domingues

**Affiliations:** 1 CEB-Centre of Biological Engineering, University of Minho, 4710-057 Braga, Portugal; 2 Metabolic Engineering Group, Department of Microbiology and Genetics, University of Salamanca, Campus Miguel de Unamuno, 37007 Salamanca, Spain

**Keywords:** Volatile organic compounds, γ-Lactones, *Ashbya gossypii*, De novo biosynthesis, Metabolic engineering, Fatty acid biosynthesis, β-Oxidation

## Abstract

**Background:**

Lactones are highly valuable cyclic esters of hydroxy fatty acids that find application as pure fragrances or as building blocks of speciality chemicals. While chemical synthesis often leads to undesired racemic mixtures, microbial production allows obtaining optically pure lactones. The production of a specific lactone by biotransformation depends on the supply of the corresponding hydroxy fatty acid, which has economic and industrial value similar to γ-lactones. Hence, the identification and exploration of microorganisms with the rare natural ability for de novo biosynthesis of lactones will contribute to the long-term sustainability of microbial production. In this study, the innate ability of *Ashbya gossypii* for de novo production of γ-lactones from glucose was evaluated and improved.

**Results:**

Characterization of the volatile organic compounds produced by nine strains of this industrial filamentous fungus in glucose-based medium revealed the noteworthy presence of seven chemically different γ-lactones. To decipher and understand the de novo biosynthesis of γ-lactones from glucose, we developed metabolic engineering strategies focused on the fatty acid biosynthesis and the β-oxidation pathways. Overexpression of *AgDES589*, encoding a desaturase for the conversion of oleic acid (C18:1) into linoleic acid (C18:2), and deletion of *AgELO624*, which encodes an elongase that catalyses the formation of C20:0 and C22:0 fatty acids, greatly increased the production of γ-lactones (up to 6.4-fold; (7.6 ± 0.8) × 10^3^ µg/g_Cell Dry Weight_). Further substitution of *AgPOX1*, encoding the exclusive acyl-CoA oxidase in *A. gossypii*, by a codon-optimized *POX2* gene from *Yarrowia lipolytica*, which encodes a specific long chain acyl-CoA oxidase, fine-tuned the biosynthesis of γ-decalactone to a relative production of more than 99%.

**Conclusions:**

This study demonstrates the potential of *A. gossypii* as a model and future platform for de novo biosynthesis of γ-lactones. By means of metabolic engineering, key enzymatic steps involved in their production were elucidated. Moreover, the combinatorial metabolic engineering strategies developed resulted in improved de novo biosynthesis of γ-decalactone. In sum, these proof-of-concept data revealed yet unknown metabolic and genetic determinants important for the future exploration of the de novo production of γ-lactones as an alternative to biotransformation processes.

**Electronic supplementary material:**

The online version of this article (10.1186/s12934-019-1113-1) contains supplementary material, which is available to authorized users.

## Background

Lactones are volatile organic compounds (VOCs) derived from the lipid metabolism. They are chemically classified as cyclic esters of hydroxy fatty acids and find application mainly as pure fragrances and flavours or as building blocks of speciality chemicals [1]. These compounds are naturally present in ripe fruits, such as peach and strawberry, and contribute extensively to their complex aroma and flavour [2, 3]. Consequently, lactones can be found in products of the food and cosmetics industries, reaching a world market volume of hundreds of tons per year [4]. The most relevant lactone commercialized is γ-decalactone, characterized by a strong peach-like odour.

Lactones are still mainly obtained by chemical synthesis [4]. However, this type of process often leads to undesired racemic mixtures, has the problem of not being well accepted by the consumers and withstands serious environmental problems linked to fossil fuels [1, 5]. Therefore, microbial production has appeared as a sustainable biotechnological alternative that offers the possibility to obtain pure lactones.

Microbial production of lactones has been accomplished through biotransformation processes, where an immediate precursor of lactones (an hydroxy fatty acid) is added to the production media to be converted into the desired lactone. The majority of the biotransformation processes explored at the industrial scale use the yeast *Yarrowia lipolytica* to convert ricinoleic acid (e.g., from castor oil) to γ-decalactone. Following the same enzymatic pathway, the change of the precursor leads to the production of a different lactone [1]. For instance, the use of 10-hydroxystearic acid results in the production of γ-dodecalactone [6]. Thus, in these processes, the production of a particular lactone entirely relies on the availability of the corresponding precursor. This is a major drawback, since these precursors often have economic value and industrial applicability equivalent to that of lactones [7]. Moreover, castor oil, the raw substrate most commonly used, is connected to health and safety issues concerning its extraction from the plant *Ricinus communis* [8].

Given all of the above, the identification and engineering of microbial systems with the unusual natural ability for de novo biosynthesis of lactones from sustainable and highly accessible carbon sources, such as carbohydrates, constitutes a major challenge to this field. To the extent of our knowledge, only three microbial species were previously studied for the de novo biosynthesis of lactones: two filamentous fungi, *Fusarium poae* [9] and *Trichoderma viride* [10, 11]; and one yeast, *Sporidiobolus salmonicolor* [12, 13]. However, a comprehensive overview of this biosynthetic process is lacking and there is very little information regarding the genetic and enzymatic machinery involved.

**Fig. 1:**
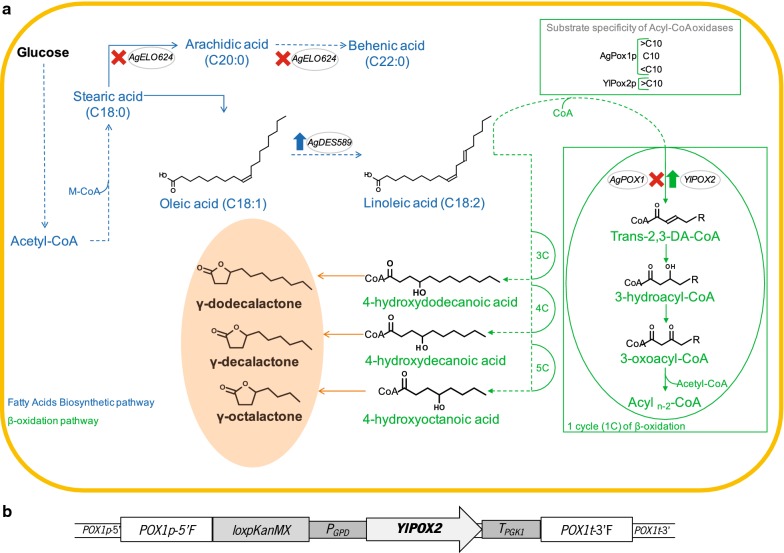
Metabolic engineering of the de novo biosynthesis of lactones in *A. gossypii*. **a** Schematic diagram of the interconnection between the metabolic pathways involved in the de novo biosynthesis of lactones. An acyl_n−2_-CoA represents a fatty acid from which two carbons of the initial chain length were removed after 1 cycle of β-oxidation. **b** Heterologous overexpression cassette constructed using the Golden-Gate rapid assembly. Grey-circled names represent the genes targeted for engineering. Dashed lines represent multi-reactions. M-CoA: malonyl-CoA; trans-2,3-DA-Coa: trans-2,3-dehydroacyl-CoA

*Ashbya gossypii* (*syn*. *Eremothecium gossypii*) is a filamentous fungus of the Saccharomycetaceae family that has been used in the industrial production of riboflavin (vitamin B_2_) for more than 20 years [14, 15] and which presents important industrial advantages [16]. Moreover, the molecular and in silico tools available for its engineering are steadily growing and omics data are becoming increasingly available [17]. Together with these technical advantages, *A. gossypii* owns a very rich and heterogeneous metabolism mainly modulated by riboflavin overproduction [18], but being able to produce many other metabolites such as aromatic VOCs [19]. In addition, since riboflavin is produced from the lipids that this fungus is able to biosynthesize [20] and to accumulate [21, 22], *A. gossypii* shows up as a suitable microbial model for studying and modulating the production of the fatty acid-derived VOCs: lactones. Therefore, in this study, the innate potential of several *A. gossypii* strains to biosynthesize lactones was evaluated and metabolic engineering strategies towards the fatty acid biosynthesis and the β-oxidation pathways were performed (Fig. 1a) with the objective of deciphering and understanding the intricate de novo biosynthesis of lactones in *A. gossypii*.

## Results and discussion

### *A. gossypii* predominantly produced γ-lactones and higher alcohols as VOCs

**Fig. 2:**
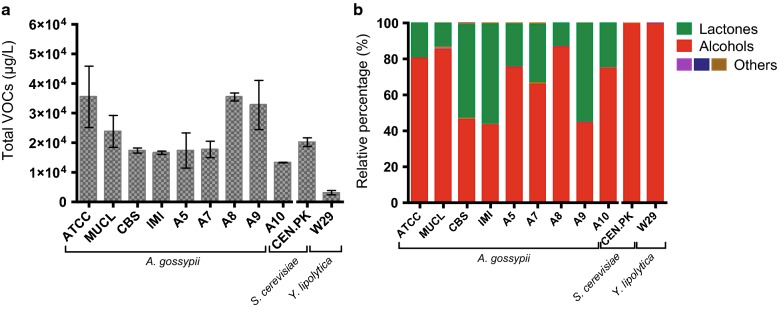
Quantitative and qualitative VOCs profiling by GC–MS of *A. gossypii* strains. **a** Total VOCs production. **b** Distribution of the VOCs produced by families of aromas according to their chemical structure and their relative presence in each strain. The *S. cerevisiae* CEN.PK113-7D (CEN.PK) and *Y. lipolytica* W29 were used as controls to help evaluate which VOCs were distinctively produced by *A. gossypii*. Cultures were carried out in MA2 medium for 48 h. Data are presented as mean ± standard deviation of at least two biological replicates

A complete metabolic profiling of the VOCs produced by *A. gossypii* was performed to understand its innate potential to biosynthesize lactones. Nine *A. gossypii* strains obtained from different collections (Table 1) were screened in glucose-based media (MA2). From this screening, it was possible to identify 31 to 50 different VOCs in each strain. Total production of VOCs was quantified (Fig. 2a) after 48 h of culture, when glucose was already depleted from the medium. By grouping these compounds according to their chemical structure into families of commercially valuable aromas, it was possible to notice that *A. gossypii* strains produced predominantly higher alcohols and γ-lactones (Fig. 2b). Higher alcohols represented 43–87% of the VOCs produced by *A. gossypii*, while γ-lactones had a relative representation of 6–57%. On the other hand, the yeasts *Saccharomyces cerevisiae* and *Y. lipolytica* demonstrated an almost exclusive production of higher alcohols and absence of γ-lactones production (Fig. 2b). These results confirm the distinctive trait of *A. gossypii* for de novo biosynthesis of γ-lactones.

**Table 1:** *A. gossypii* strains used and generated in this work

*A. gossypii* strains	Relevant genotype	Parental strain	References
ATCC 10895	Sequenced strain; non-engineered	–	Prof. P. Philippsen, (University of Basel)
MUCL 29450	Non-engineered	–	[23]
IMI 31268	Non-engineered	–	[23]
CBS 109.26	Non-engineered	–	[23]
A5	Non-engineered	–	[24]
A7	Non-engineered	–	[24]
A8	Non-engineered	–	[24]
A9	Non-engineered	–	[24]
A10	Non-engineered	–	[24]
DES589	*P*_*GPD*_-*AgDES589*	ATCC 10895	[27]
elo624Δ	*Agelo624Δ*	ATCC 10895	[27]
DES589/elo624Δ	*P*_*GPD*_-*AgDES589, Agelo624Δ*	DES589	This work
YlPOX2	*Agpox1Δ::loxP*-*KanMX4*-*loxP*-*P*_*GPD*_-*synYlPOX2*	ATCC 10895	This work
DES589/elo624Δ/YlPOX2	*P*_*GPD*_-*AgDES589, Agelo624Δ, Agpox1Δ::loxP*-*KanMX4*-*loxP*-*P*_*GPD*_-*synYlPOX2*	DES589/elo624Δ	This work

Seven chemically different γ-lactones were identified during the growth phase of this fungus (from 24 h onward), namely γ-valerolactone (PubChem CID: 7921), γ-caprolactone (PubChem CID: 12756), γ-octalactone (PubChem CID: 7704), γ-nonalactone (PubChem CID: 7710), γ-decalactone (PubChem CID: 12813), γ-undecalactone (PubChem CID: 7714) and γ-dodecalactone (PubChem CID: 16821). All the γ-lactones here detected were identified using pure standards. Their identification was also confirmed by the presence of the molecular ion peak of *m/z* 85 in the MS spectrum, which is characteristic of γ-lactones as listed in the National Institute of Standards and Technology (NIST). In addition, it can be assumed that *A. gossypii* did not produce δ-lactones since the molecular ion peak of *m/z* 99, representative of δ-lactones, was not identified in any of the MS spectra (Table 2). When compared with other microorganisms, the chemical diversity of the γ-lactones biosynthesized by *A. gossypii* and the relative short time needed for their production (2 days) is noteworthy. Only three (*T. viride*; 12 days) [11], four (*S. salmonicolor*; 12 days) [12] and five (*F. poae*; 8 days) [9] γ-lactones were detected in the other reported lactone-producing fungi. Regarding the other predominant family of VOCs, the two major higher alcohols detected were 2-phenylethanol and isoamyl alcohol (Additional file 1: Fig. S1), which is in accordance with data previously reported for the ATCC 10895 strain [19].

**Table 2:** MS fragmentation pattern for each γ-lactone

Compound	Characteristic ions
γ-Valerolactone	56 (100) + 85 (69) + 41 (64) + 39 (56) + 43 (38)
γ-Caprolactone	85 (100)+ 57 (25) + 39 (17) + 55 (16) + 56 (13)
γ-Octalactone	85 (100) + 57 (22) + 39 (14) + 41 (11) + 55 (9)
γ-Nonalactone	85 (100)+ 57 (20) + 39 (16) + 41 (13) + 55 (11)
γ-Decalactone	85 (100)+ 57 (20) + 39 (16) + 41 (15) + 55 (15)
γ-Undecalactone	85 (100)+ 57 (27) + 41 (19) + 55 (16) + 128 (12)
γ-Dodecalactone	85 (100) + 39 (24) + 57 (24) + 55 (24) + 41 (23)

Numbers between brackets represent percentages of m/z ions

### The major lactone produced by *A. gossypii* was γ-decalactone

**Fig. 3:**
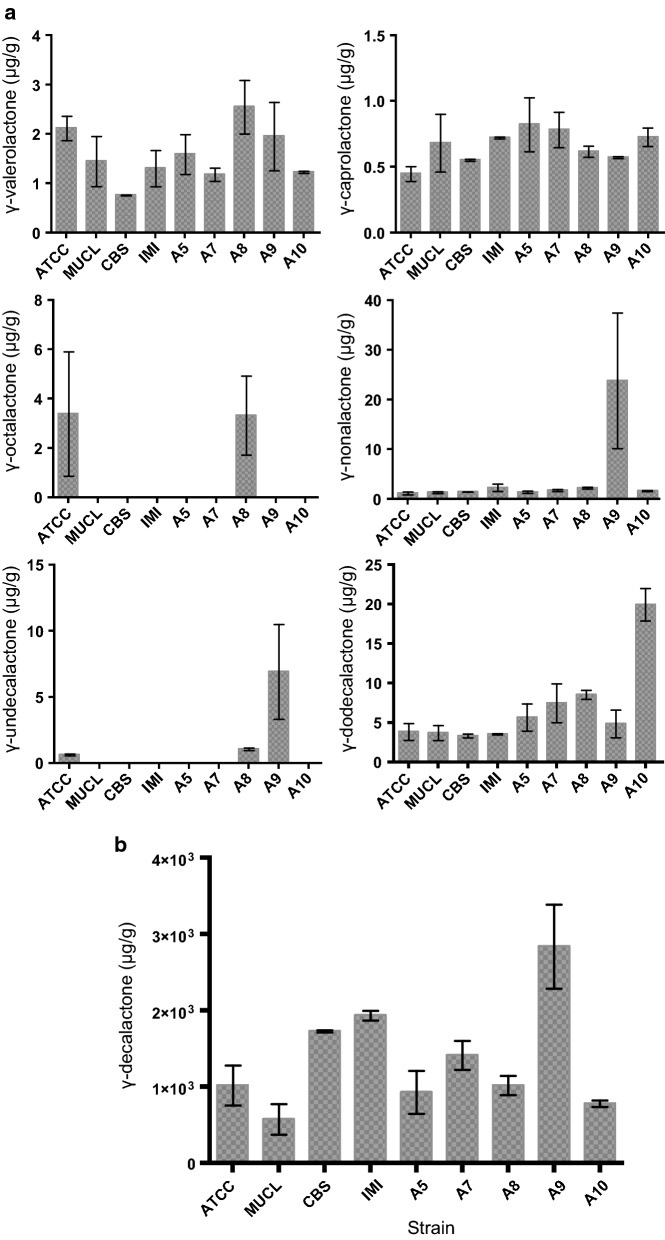
Specific productions (μg/g_CDW_) of the seven chemically different γ-lactones identified in cultures of *A. gossypii*. **a** Production levels of the minor γ-lactones identified. **b** Production levels of the major γ-lactone identified, γ-decalactone. Data are presented as mean ± standard deviation of at least two biological replicates

The distinctive nature of *A. gossypii* for de novo biosynthesis of γ-lactones from glucose was extensive to all strains tested. However, among the seven γ-lactones biosynthesized, one was clearly produced with major prevalence, γ-decalactone. Compared to the other six γ-lactones (Fig. 3a), γ-decalactone was produced in concentrations two orders of magnitude higher, reaching (2.8 ± 0.6) × 10^3^ µg/g_CDW_ (Fig. 3b). Regarding the six minor γ-lactones detected, their concentration in the medium was not uniformly distributed, being γ-dodecalactone the most representative among them (Fig. 3a). On the other hand, the specific production of γ-lactones was heterogeneous among the different strains (Fig. 3). For instance, the best γ-decalactone-producing strain (concerning average specific production) was strain A9, while strain A10 was superior for γ-dodecalactone biosynthesis. Strain heterogeneity in *A. gossypii* was previously reported regarding growth rates in different carbon sources [23] and also regarding nucleosides production [24]. Therefore, when investigating *A. gossypii* for the purpose of its development as a cell factory, the exploration of this specie’s biodiversity gives a more comprehensive understanding of its potential.

Noteworthy, four of the seven γ-lactones produced by *A. gossypii* were detected in volumetric concentrations above their odour perception threshold (Additional file 1: Table S1) [25, 26]. Therefore, these active fragrances possibly contribute to the characteristic aroma released from the cultures of this fungus. Specifically, γ-decalactone seems to be the major contributor among the fragrances produced from the *A. gossypii* strains tested.

Since these results revealed a yet unknown potential of *A. gossypii* for de novo biosynthesis of γ-lactones from sugar-based media, the native pathways involved in γ-lactone formation merited further elucidation.

### Redirection of the metabolic fluxes of the fatty acid biosynthesis modulated the de novo biosynthesis of γ-lactones in *A. gossypii*

Lactones are VOCs derived from the fatty acid metabolism. In natural producers, such as peach (*Prunus persica* L. Batsch) or strawberry (*Fragaria* × *ananassa*) fruits, the direct precursors for lactones biosynthesis are unsaturated fatty acids [2, 3]. In *A. gossypii*, more than 80% of the total fatty acids accumulated during growth in sugar-based media are unsaturated [22]. Of these, oleic acid (C18:1) represents more than 50% of the total fatty acid composition [27]. A first experimental indication that oleic acid could positively contribute for the biosynthesis of lactones in *A. gossypii* was obtained from fermentations where glucose was substituted by oleic acid as the carbon source. While the production of γ-decalactone remained similar, oleic acid stimulated the biosynthesis of some minor lactones, such as γ-dodecalactone, which in this condition was produced in amounts 100-fold higher than in glucose-based medium (data not shown). These data indicated oleic acid as an important precursor for the de novo biosynthesis of γ-lactones and is in good agreement with the fact that oleic acid is the starting point for the production of γ-dodecalactone in biotransformation processes [6]. Furthermore, oleic acid was identified as a central intermediate in the biosynthesis of lactones in the yeast *S. salmonicolor* [13].

Thus, to confirm this hypothesis and to assess whether the biosynthesis of γ-lactones from glucose could be improved through the redirection of the oleic acid metabolic flux in the *A. gossypii’s* fatty acid biosynthetic pathway, two genetic modifications were designed: (i) the overexpression of the gene *AgDES589*, encoding a desaturase for the conversion of oleic acid into linoleic acid (EC 1.14.19.6); and (ii) the deletion of the gene *AgELO624*, encoding an elongase that catalyses the formation of fatty acids of C20:0 and C22:0 (EC 2.3.1.199) (Fig. 1a). It is worth of noting that the fatty acid profile of *A. gossypii* is characterized by the presence of 4–8% of fatty acids longer than C18 [22, 27]. The background chosen for the genetic modifications was the best characterized and sequenced strain ATCC 10895 (ATCC).

**Fig. 4:**
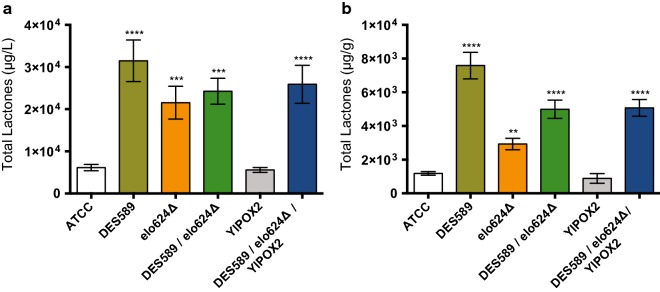
Improved biosynthesis of γ-lactones by engineered *A. gossypii* strains. **a** Volumetric (μg/L) and **b** specific (μg/g_CDW_) production of total γ-lactones. Data are presented as mean ± standard deviation of three biological replicates. Cultures were carried out in AFM for 72 h. Asterisks represent significant differences to the control (ATCC). **(*p* < 0.01), ***(*p* < 0.001) and ****(*p* < 0.0001)

The engineered strains were tested in AFM sugar-based medium. The transition from MA2 to AFM is justified by superior biomass production obtained with this medium that led to a higher volumetric production of γ-lactones (~ tenfold; Fig. 4a). The time point chosen for the comparisons was 72 h, when all the glucose was already depleted from the medium, because a small but increasing trend in the production of γ-lactones by the ATCC strain was observed between 24 and 72 h (data not shown). In comparison with the parental strain, the overexpression of *AgDES589* led to the most pronounced improvement, with a 6.4-fold increase in the specific production of γ-lactones (µg/g_CDW_; Fig. 4b). However, this overexpression did not increase evenly the production of all the γ-lactones, being this strain (DES589) significantly enriched mainly in γ-octalactone and γ-decalactone, and slightly enriched in γ-dodecalactone (Table 3). *AgDES589* was previously shown to be transcriptionally inhibited by the product (linoleic acid) [27]. In addition, enzyme feedback regulation by products was reported for other desaturases [27]. Therefore, gene and/or protein engineering of AgDes589p to eliminate potential regulatory mechanisms could further potentiate the positive effect of this enzyme on the biosynthesis of lactones in *A. gossypii*.

**Table 3:** Specific production (μg/g_CDW_) of different γ-lactones by engineered *A. gossypii* strains

γ-Lactones	ATCC	DES589	elo624Δ	DES589/elo624Δ	YlPOX2	DES589/elo624Δ/YlPOX2
γ-Octalactone	–	(1.3 ± 0.2)^a^ × 10^2^	–	(1.1 ± 0.1)^a^ × 10^2^	–	(3.2 ± 1.0)^d^ × 10^1^
γ-Nonalactone	–	6 ± 2	–	3 ± 0	–	–
γ-Decalactone	(1.2 ± 0.1)^a^ × 10^3^	(7.4 ± 0.8)^e^ × 10^3^ 98.0 ± 0.2 ^a^	(2.9 ± 0.3)^c^ × 10^3^	(4.9 ± 0.5)^e^ × 10^3^ 97.7 ± 0.2 ^a^	(8.8 ± 2.9)^a^ × 10^2^	(5.0 ± 0.5)^e^ × 10^3^ 99.3 ± 0.1 ^e^
γ-Undecalactone	–	3 ± 1	–	2 ± 0	–	2 ± 1
γ-Dodecalactone	3 ± 1^a^	(1.4 ± 0.3)^b^ × 10^1^	2 ± 0^a^	3 ± 0^a^	3 ± 1^a^	2 ± 1^a^

Underlined values represent the percentage (%) of γ-decalactone regarding the total γ-lactones produced by different high-producing engineered strains. Data are presented as mean ± standard deviation of three biological replicates. Superscript letters represent significant differences to the control (ATCC) between values of the same column. Superscript letters of underlined values represent significant differences between relative productions. ^a,b^(*p* < 0.05), ^a,c^(*p* < 0.01), ^a,d^(*p* < 0.001) and ^a,e^(*p* < 0.0001)

(–), not detected and considered 0 for the statistical tests

In turn, strain elo624Δ produced 2.5-fold more lactones, specifically γ-decalactone. This result suggests that very long fatty acids (C20 to C26) are not ideally used as precursors for lactones biosynthesis. This may be related with their saturated nature. Even if shortened in the β-oxidation pathway, these fatty acids would not become an appropriate substrate for AgDes589p. Moreover, as the other elongase encoded in the *A. gossypii* genome (AgElo586p) is able to elongate fatty acids of C18:0 and C20:0 with poor efficiency [27], its deletion would be expected to further improve lactones’ biosynthesis.

Finally, combination of the two genetic modifications in strain DES589/elo624Δ resulted in a 4.2-fold increase in lactone production, reflected exclusively in the two main γ-lactones produced by strain DES589, γ-octalactone and γ-decalactone (Fig. 4b; Table 3).

Together, these results clearly show that the deregulation of the fatty acid biosynthetic pathway at the C18 level significantly modulated the biosynthesis of γ-lactones in *A. gossypii*. Oleic acid, the main fatty acid accumulated by *A. gossypii*, is a central metabolite. As shown in Table 3, the biosynthesis of γ-lactones increased upon blockage of its conversion into longer fatty acids (strain elo624Δ). Moreover, before entering the β-oxidation pathway, oleic acid is very likely to undergo additional chemical modifications. In accordance, the overexpression of the gene *AgDES589*, which promotes the desaturation of oleic acid (C18:1) into linoleic acid (C18:2) (Fig. 1), was shown to strongly enhance the biosynthesis of γ-lactones. In strawberry fruit, the expression of the gene *FaFAD1*, encoding a Δ12 fatty acid desaturase, was positively correlated with the γ-decalactone content in fruits [3]. A homologue of *FaFAD1* in peach fruit was also connected with lactone biosynthesis [2]. A BLAST^®^ (https://blast.ncbi.nlm.nih.gov/Blast.cgi) retrieved *FaFAD1* as an ortholog of *AgDES589*. AgDes589p shares 39% amino acid identity with *Fa*Fad1p (Additional file 1: Fig. S2). In contrast, no orthologs of the *AgDES589* gene were found in the closely related yeast *S. cerevisiae* or in other post-WGD species of the Saccharomycetaceae. Therefore, *AgDES589* is one of the underlying genetic differences conferring *A. gossypii* the ability for de novo biosynthesis of γ-lactones.

Interestingly, and just as FaFad1p, two amino acid residues important for the catalytic activity [3] are conserved between AgDes589p and hydroxylases (e.g., *R. comunis* RcFah12p) or bifunctional hydroxylase/desaturases (*Lesquerella fendleri* bifunctional LfAh12p), rather than with exclusive desaturases (e.g., *Arabidopsis thaliana* AtFad2p). This suggests that AgDes589p could be an enzyme with both desaturase and hydroxylase activity. *Y. lipolytica*, the workhorse used for the production of γ-decalactone by biotransformation, also owns a protein similar to AgDes589p (Additional file 1: Fig. S2). However, this enzyme is only conserved in one of the amino acids critical for the hydroxylase activity. Not owning enzyme(s) able to catalyse the formation of hydroxy fatty acids from oleic acid could be the reason why the production of γ-decalactone by this yeast is only reported from hydroxy fatty acids (e.g., ricinoleic acid) [1], despite the major accumulation of oleic acid inside the cells [28]. Thus, studying the activities of these enzymes is of pivotal importance to further understand their role in the de novo biosynthesis of lactones.

In fruits [2, 3], the biosynthesis of γ-lactones has been proposed to start from the hydration or hydroxylation of unsaturated fatty acids. Considering our data, we propose that the cellular pools of oleic acid (18:1), naturally synthesized and accumulated by *A. gossypii* [22], are directed to linoleic acid formation (18:2) by the introduction of an additional unsaturation (Fig. 1). In turn, linoleic acid should be hydrated to form hydroxy fatty acids by a yet unidentified enzyme, similarly to what was proposed for fruits [2, 3]. As mentioned above, we cannot exclude that AgDes589p may also possess hydroxylase activity and therefore be able to catalyse the conversion of oleic acid into hydroxy fatty acids. These lactones’ precursors are then shortened at the β-oxidation pathway, generating 4-hydroxy acids of different lengths. Finally, it is widely accepted that γ-lactones are formed by spontaneous lactonisation [1–3] (Fig. 1).

### Combinatorial engineering fine-tuned the biosynthesis of γ-decalactone in *A. gossypii*

Pure lactones obtained by microbial production are preferred instead of racemic mixtures, because the former facilitates downstream processing [1]. The operations used for that end have been liquid–liquid two-phase extraction and distillation, which are designed to purify one lactone [29, 30]. Since the application as pure fragrances is the most relevant route for their commercialization, fine-tuning the biosynthesis of γ-lactones towards a particular one and therefore create tailored engineered strains is envisaged. Hence, the *AgPOX1* gene was replaced by a heterologous overexpression cassette containing a *POX2* gene from *Y. lipolytica* (Fig. 1a). *AgPOX1* codifies for the exclusive acyl-CoA oxidase (EC 1.3.3.6) encoded in the *A. gossypii* genome that catalyses the first reaction of the β-oxidation pathway [22], while *YlPOX2* codifies for a specific long chain acyl-CoA oxidase (EC 1.3.3.6) with activity for C11–C18 fatty acids [31]. With this modification, the degradation of fatty acids in the β-oxidation pathway should stop at the C10 level and consequently the biosynthesis of lactones shorter than γ-decalactone would be prevented. Furthermore, this strategy would confirm the role of the β-oxidation pathway in the de novo biosynthesis of lactones by *A. gossypii*.

The overexpression of *YlPOX2* did not produce any significant effect on the total production (Fig. 4) nor on the profile of γ-lactones biosynthesized (Table 3) as compared with the parental strain. However, fine-tuning was accomplished by combining the three genetic modifications in strain DES589/elo624Δ/YlPOX2. This strain presents a similar production of γ-decalactone and lower production of γ-octalactone when compared to strains DES589 and DES589/elo624Δ (Table 3). Noteworthy, the percentage of γ-decalactone produced by this strain was significantly higher than that obtained with the other two overproducer strains, DES589 and DES589/elo624Δ (Table 3). This fine-tuning occurred at an extent that γ-decalactone represented 99.3% of the total γ-lactones produced, confirming that the specificity of the acyl-CoA oxidase in the β-oxidation contributes to the qualitative profile of the final product.

## Conclusions

An efficient microorganism for the de novo biosynthesis of lactones should: (i) present a superior ability to transform carbohydrates into fatty acids; (ii) possess the genetic machinery for the conversion of the latter into lactones. Here, we show that *A. gossypii* fulfils these two requisites. Taken together, these results indicate that the fatty acid biosynthesis and the β-oxidation are the central pathways involved in the de novo biosynthesis of γ-lactones in *A. gossypii*. Regarding the first, *A. gossypii* has been successfully engineered to synthesize biolipids not only from hexoses [32], but also from pentoses [33], expanding its potential as a model for studying the biosynthesis of lactones from different sugars.

Moreover, by means of metabolic engineering, this work elucidates key enzymatic steps for de novo biosynthesis of γ-lactones in *A. gossypii* and shows how this can be further improved and fine-tuned. To the extent of our knowledge, this is the first example of improved de novo biosynthesis of γ-lactones achieved by an engineered microorganism. The realization of microbial de novo biosynthesis of γ-lactones is of upmost importance, paving the way for the sustainable production of these valuable compounds with a broad range of biological or odorant properties.

## Materials and methods

### Chemicals

The following chemicals with the corresponding purity were used as standards for the gas chromatography-mass spectrometry (GC–MS) analysis: 3-methyl-1-butanol (≥ 99.8%), 2-phenylethanol (≥ 99%) and γ-octalactone (> 98%) from Fluka; γ-valerolactone (> 98%) from TCI; γ-caprolactone (> 98%), γ-nonalactone (> 98%), γ-decalactone (≥ 98%), γ-dodecalactone (≥ 97%) and γ-undecalactone (≥ 98%) from Sigma-Aldrich.

### Strains and media

The *A. gossypii* strains used and generated during this study are listed in Table 1. These strains were maintained on agar-solidified AFM (10 g/L yeast extract, 10 g/L tryptone, 1 g/L myo-inositol and 20 g/L glucose) or agar-solidified MA2 medium (20 g/L peptone, 2 g/L yeast extract, 0.6 g/L myo-inositol and 20 g/L glucose, pH 6.8). Spores from *A. gossypii* were isolated and stored as described previously [34]. The *S. cerevisiae* CEN.PK113-7D (MAT α, MAL2-8c, SUC2; INSA, France) and *Y. lipolytica* W29 (ATCC 20460:CLIB89) used for comparative purposes during the screening of the volatile compounds (VOCs) were maintained on agar-solidified YPD medium (10 g/L yeast extract, 20 g/L peptone and 20 g/L glucose). *Escherichia coli* DH5α strain served as host for the cloning experiments and was grown on agar-solidified LB medium (10 g/L tryptone, 5 g/L yeast extract and 10 g/L NaCl, pH 7.0) containing, when appropriate, 100 µg/mL ampicillin, 50 µg/mL kanamycin or 100 µg/mL spectinomycin for the selection and maintenance of plasmids.

### Culture conditions for the analysis of the VOCs produced by *A. gossypii* non-engineered strains

As not all the *A. gossypii* strains are able to sporulate under laboratory conditions [23], the inocula for the screening of VOCs were made as follows. Grown mycelium of each strain was scrapped off an agar-solidified AFM plate and digested with 4 mg/mL lysing enzymes from *Trichoderma harzianum* (Sigma-Aldrich) in order to obtain more homogeneously dispersed mycelium. Subsequently, this was used to prepare a pre-inoculum in MA2 medium that was grown for 48 h in order to obtain a reproducible and normalized amount of mycelium for all strains. Finally, 1 mL of this pre-inoculum was used to inoculate 250 mL shake-flasks with 50 mL of MA2 medium. *A. gossypii* cultures were carried out at 28 °C and 200 rpm. After 48 h, the total culture broth was separated by vacuum filtration using Advantec Qualitative Filter Paper (grade 2). The filtrates were immediately filtered again through 0.2 μm membrane filters and stored at − 20 °C until GC–MS analysis. The mycelium retained in the filter paper was washed with distilled water and used for cell dry weight (CDW) determination. CDW was then measured by collecting the mycelium into a pre-weighed dried tube and drying at 105 °C until constant weight (~ 24 h).

### Construction of integration cassettes and transformation of *A. gossypii*

DNA manipulations were made using standard molecular biology procedures [35]. Different transformation cassettes were constructed either for the deletion of native genes or for the overexpression of heterologous genes. For the deletion of the *AgELO624* gene (NCBI Reference Sequence: NM_212307.1), the entire open reading frame (ORF) was replaced by a cassette comprising a *loxP*-*KanMX*-*loxP* marker that was obtained by PCR amplification using specific primers (Additional file 1: Table S2). These primers were used to introduce recombinogenic flanks for the *AgELO624* ORF in both sides, corresponding to regions upstream the start codon and downstream the stop codon of the gene. For the overexpression of the heterologous *POX2* gene from *Y. lipolytica* (NCBI Reference Sequence: XM_505264.1), the ORF was codon-optimized for the *A. gossypii* codon usage. This gene was obtained from Integrated DNA Technologies (IDT^®^). This synthetic gene was then amplified using specific primers (Additional file 1: Table S2) and subsequently cloned in a pBluescript SK (−) vector. Besides the codon-optimized *YlPOX2* gene, the heterologous overexpression cassette contained two recombinogenic flanks, a selection marker *loxP*-*KanMX*-*loxP*, and the regulatory *AgGPD* promoter and *AgPGK1* terminator sequences (Fig. 1b). The recombinogenic flanks of this cassette were targeted to *AgPOX1* locus. Thus, the entire ORF of the *AgPOX1* gene (NCBI Reference Sequence: NM_210568.2) was completely replaced by the *YlPOX2* overexpression cassette. The *AgPOX1* recombinogenic flanks were also PCR-amplified using specific primers (Additional file 1: Table S2) and cloned in a pBluescript SK (−) vector. The modules comprising the selection marker and the regulatory sequences were amplified and cloned previously [22]. Finally, all the modules of the overexpression cassette were verified by DNA sequencing and assembled together with the acceptor vector using the *Bsa*I restriction enzyme, accordingly with the Golden-Gate method previously described [36, 37]. Before transformation in *A. gossypii*, the heterologous overexpression cassette (Fig. 1b) was linearized by enzymatic restriction with *Sap*I.

Spores of *A. gossypii* ATCC 10895 were transformed with the corresponding cassettes using the Cre-*lox*P system [38], which enabled the elimination and reuse of the selection marker through the expression of a Cre recombinase in *A. gossypii* as described previously [39]. The primary heterokaryotic transformants were selected on agar-solidified MA2/AFM containing 250–300 μg/L geneticin (G418). Selection of *A. gossypii* homokaryotic clones was performed through the germination of uninucleated haploid spores obtained from the primary heterokaryotic transformants on agar-solidified MA2/AFM-G418. The correct genomic integration of each cassette as well as the homokaryotic genotype of each clone were confirmed by analytical PCR and DNA sequencing. For this purpose, a pin-head of mycelium from single colonies was suspended in 30 μL of extraction buffer (0.05 M carbonate buffer pH 6.9, 2% (w/v) PVP 40, 0.2% (w/v) BSA and 0.05% (v/v) Tween 20) and incubated for 10 min at 100 °C. Afterwards, the tubes were centrifuged at maximum speed. The supernatant was used as template in the PCR reactions using the specific primers listed in Additional file 1: Table S2.

### Culture conditions for the analysis of the de novo biosynthesis of γ-lactones by *A. gossypii* engineered strains

The cultures of the engineered strains were initiated with the inoculation of 10^7^ spores in 50 mL of AFM and carried out in 250 mL shake-flasks at 28 °C and 200 rpm. After 72 h, the mycelium and filtrate were collected and treated as described above.

### Volatiles extraction and analysis by GC–MS

Volatiles were analysed by GC–MS after extraction from 8 mL samples with 400 µL of dichloromethane, with 4-nonanol as internal standard. A gas chromatograph Varian 3800 with a 1079 injector and an ion-trap mass spectrometer Varian Saturn 2000 was used. 1 µL injections were made in splitless mode (30 s) in a Sapiens-Wax MS column (30 m; 0.15 mm; 0.15 µm film thickness, Teknokroma). Carrier gas was helium 49 (Praxair) at a constant 1.3 mL/min flow. Detector was set to electronic impact mode with an ionization energy of 70 eV, a mass acquisition range from 35 to 260 m/z and 610 ms acquisition interval. Oven temperature was initially set to 60 °C for 2 min and then raised from 60 to 234 °C at a rate of 3 °C/min, raised from 234 to 260 °C at 5 °C/min and finally maintained at 260 °C for 10 min. Injector temperature was 250 °C with 30 mL/min split flow. Compounds were identified using MS Workstation version 6.9 (Varian) software, by comparing mass spectra and retention indices with those of pure standards mentioned above. Volatiles were quantified as 4-nonanol equivalents.

### Statistical analyses

GraphPad Prism for IOS version 6.0 was used to carry out the statistical analyses. Differences to a control strain were analysed by one-way ANOVA followed by Dunnett’s multiple comparison test. When no control strain is indicated, the analyses were made by one-way ANOVA followed by Tukey’s multiple comparison test. Unless indicated otherwise, statistical significance was established at *p *< 0.05 for the comparisons.

## Additional file

**Additional file 1: Fig. S1.** Specific productions (μg/g_CDW_) of the two major higher alcohols identified in cultures of non-engineered *A. gossypii* strains. Production levels of (A) 2-phenylethanol and (B) isoamyl alcohol. Data are presented as mean ± standard deviation of at least two biological replicates. **Table S1.** Lactones produced by *A. gossypii* non-engineered strains above their odour perception threshold. Values of the thresholds either in complex (*e.g.*, wine) or simple (*e.g.*, aqueous solutions) samples were retrieved from the literature [25, 26]. Data are presented as mean of at least two biological replicates. (−), not detected or detected at concentrations below the odour perception threshold of the compound. **Fig. S2.** Comparison of the amino acid sequence of *A. gossypii* AgDes589p with other fatty acid desaturases, hydroxylases and bifunctional desaturase/hydroxylases. Asterisks (*) represent conserved residues, colons (:) represent residues with strong similar properties and periods (.) represent residues with weak similar properties. The amino acid residues that differ between oleate desaturases and hydroxylases are indicated in boxes. Red boxes represent the two important amino acids residues for the catalytic activity that are conserved between AgDes589p and hydroxylases or bifunctional enzymes. Accession numbers for the sequences are: *F.* x *ananassa* desaturase FaFAD1p (KF887973), *Y. lipolytica* YlYALI0B10153p (XP_500707.1), *A. thaliana* AtFAD2p (AAA32782), *R. comunis* hydroxylase RcFAH12 (AAC49010), (MDP0000288297) and *L. fendleri* bifunctional desaturase/hydroxylase LFAH12 (AAC32755). **Table S2.** Primers used in this work. Lower case sequences correspond to homologous recombination sites. Recognition sequences for restriction enzymes are underlined.

## Abbreviations

VOCs: volatile organic compounds
ORF: open reading frames
