# Supplementary material for: Metabolic engineering of Ashbya gossypii for deciphering the de novo biosynthesis of γ-lactones
Source: Microb Cell Fact. 2019 Mar 28;18:62. doi: 10.1186/s12934-019-1113-1 (PMC6437850; doi:10.1186/s12934-019-1113-1)
Supplement: Supplementary file 1 — Additional file 1: Fig. S1. Specific productions (μg/gCDW) of the two major higher alcohols identified in cultures of non-engineered A. gossypii strains. Production levels of (A) 2-phenylethanol and (B) isoamyl alcohol. Data are presented as mean ± standard deviation of at least two biological replicates. Table S1. Lactones produced by A. gossypii non-engineered strains above their odour perception threshold. Values of the thresholds either in complex (e.g., wine) or simple (e.g., aqueous solutions) samples were retrieved from the literature [25, 26]. Data are presented as mean of at least two biological replicates. (−), not detected or detected at concentrations below the odour perception threshold of the compound. Fig. S2. Comparison of the amino acid sequence of A. gossypii AgDes589p with other fatty acid desaturases, hydroxylases and bifunctional desaturase/hydroxylases. Asterisks (*) represent conserved residues, colons (:) represent residues with strong similar properties and periods (.) represent residues with weak similar properties. The amino acid residues that differ between oleate desaturases and hydroxylases are indicated in boxes. Red boxes represent the two important amino acids residues for the catalytic activity that are conserved between AgDes589p and hydroxylases or bifunctional enzymes. Accession numbers for the sequences are: F. x ananassa desaturase FaFAD1p (KF887973), Y. lipolytica YlYALI0B10153p (XP_500707.1), A. thaliana AtFAD2p (AAA32782), R. comunis hydroxylase RcFAH12 (AAC49010), (MDP0000288297) and L. fendleri bifunctional desaturase/hydroxylase LFAH12 (AAC32755). Table S2. Primers used in this work. Lower case sequences correspond to homologous recombination sites. Recognition sequences for restriction enzymes are underlined. [file 12934_2019_1113_MOESM1_ESM.docx]

**ADDITIONAL INFORMATION**

**Fig. S1** Specific productions (μg/g_CDW_) of the two major higher alcohols identified in cultures of non-enginereed *A. gossypii* strains. Production levels of (A) 2-phenylethanol and (B) isoamyl alcohol. Data are presented as mean ± standard deviation of at least two biological replicates.

**Table S1** Lactones produced by *A. gossypii* non-enginereed strains above their odour perception threshold. Values of the thresholds either in complex (*e.g.*, wine) or simple (*e.g.*, aqueous solutions) samples were retrieved from the literature (Coelho et al., 2015; Jackson et al., 2009). Data are presented as mean of at least two biological replicates. (–), not detected or detected at concentrations below the odour perception threshold of the compound.

| **Strain** | **γ-octalactone (1–67 μg/L)** | **γ-nonalactone (25 μg/L)** | **γ-decalactone (0.088–1 mg/L)** | **γ-dodecalactone (7 μg/L)** |
| --- | --- | --- | --- | --- |
| ATCC | (24.34) | – | (6.89) | (26.12) |
| MUCL | – | – | (2.83) | (17.59) |
| CBS | – | – | (8.95) | (17.45) |
| IMI | – | – | (9.19) | (16.96) |
| A5 | – | – | (4.05) | (24.9) |
| A7 | – | – | (5.71) | (29.30) |
| A8 | (15.09) | – | (4.61) | (38.75) |
| A9 | – | (151.39) | (17.74) | (30.78) |
| A10 | – | – | (3.21) | (82.07) |

Coelho, E., Vilanova, M., Genisheva, Z., Oliveira, J.M., Teixeira, J.A., and Domingues, L. (2015) Systematic approach for the development of fruit wines from industrially processed fruit concentrates, including optimization of fermentation parameters, chemical characterization and sensory evaluation. *LWT - Food Sci. Technol. 62,* 1043–1052.

Jackson, R.S. (2009) Olfactory Sensations. In: Wine Tasting: A Professional Handbook, 2nd ed. Academic Press: Burlington, San Diego, London.

**Fig. S2.** Comparison of the amino acid sequence of *A. gossypii* AgDes589p with other fatty acid desaturases, hydroxylases and bifunctional desaturase/hydroxylases. Asterisks (*) represent conserved residues, colons (:) represent residues with strong similar properties and periods (.) represent residues with weak similar properties. The amino acid residues that differ between oleate desaturases and hydroxylases are indicated in boxes. Red boxes represent the two important amino acids residues for the catalytic activity that are conserved between AgDes589p and hydroxylases or bifunctional enzymes. Accession numbers for the sequences are: *F.* x *ananassa* desaturase FaFAD1p (KF887973), *Y. lipolytica* YlYALI0B10153p (XP_500707.1), *A. thaliana* AtFAD2p (AAA32782), *R. comunis* hydroxlase RcFAH12 (AAC49010), (MDP0000288297) and *L. fendleri* bifunctional desaturase/hydroxylase LfAH12 (AAC32755).

**Table S2** Primers used in this work. Lower case sequences correspond to homologous recombination sites. Recognition sequences for restriction enzymes are underlined.

| **Primers** | **Sequence (5’– 3’)** | **Purpose** |
| --- | --- | --- |
| ELO624W-del5 | gttagttcaacataagtaagcgttacaccactgcggaagccgttattcataactccatataactgtctgagtttggtgcttcaatatttacctcaatatgcGGATCCCCGGGTTAATTAA | *AgELO624* deletion |
| ELO624W-del3 | gagtatgtaacagcgccaaacaagatctagaagattaaactaccattagaggaagagtttatcgatctatccaacatatataataacgtaatcagagttaGAATTCGAGCTCGTTTAAAC | *AgELO624* deletion |
| elo624-fw | ACTCTGCCAAGTATGCACTCCGT | *AgELO624Δ*/homok^2^  confirmation |
| kanB-rv | CTGCAGCGAGGAGCCGTAAT | *AgELO624Δ/* *YlPOX2* overex^1^ confirmation |
| elo624-rv | CGCTCTTCTGCAGCAATGCTC | *AgELO624Δ*/homok^2^ confirmation |
| (GATG)-YlPOX2-fw | TTTGGTCTCAGATGAACCCCAACAACACTGG | *YlPOX2* overex^1^ |
| (TAGT)-YlPOX2-rv | AAAGGTCTCAACTATTCCTCATCAAGCTCGCA | *YlPOX2* overex^1^ |
| (ACAC)-AgPOX1L-fw | TTTGGTCTCAACACAGTAGCGAAACACCCGAGAA | *YlPOX2* overex^1^ |
| (ATCA)-AgPOX1L-rv | TTTGGTCTCATGATCTTGTCGATCCTTGGCTGCT | *YlPOX2* overex^1^ |
| (ACGC)-AgPOX1R-fw | TTTGGTCTCAACGCTTGAGTCTATCTCTGCTCGCTG | *YlPOX2* overex^1^ |
| (CTTG)-AgPOX1R-rv | TTTGGTCTCACAAGTAGCGAGAATATGCTAGAGGTACG | *YlPOX2* overex^1^ |
| YlPOX2-b-fw | TGGGCCATGGCTCCAACCTG | *YlPOX2* sequencing |
| YlPOX2-c-rv | GGACCTTGGCGGCCTGGAAT | *YlPOX2* sequencing |
| POX1-a3-fw | GCCCGGGTAACCAAACCTTA | *YlPOX2* overex^1^/ homok^2^ confirmation |
| POX1-a3_rv | CTTGCGTGCCAGCTTAATGTC | *YlPOX2* homok^2^ confirmation |

^1^*YlPOX2* overex. refers to the construction of the *YlPOX2* overexpression module. ^2^Homokaryotic genotype.
